# Supplementary material for: Curvature flows, scaling laws and the geometry of attrition under impacts
Source: Sci Rep. 2021 Oct 19;11:20661. doi: 10.1038/s41598-021-00030-1 (PMC8526698; doi:10.1038/s41598-021-00030-1)
Supplement: Supplementary file 1 — Supplementary Information. [file 41598_2021_30_MOESM1_ESM.pdf]

Supplementary information for

G. Pál[1,2], G. Domokos[3,4], and F. Kun[1,*]

How curvature flows: scaling laws and global geometry of impact induced attrition processes

[1]Department of Theoretical Physics, Doctoral School of Physics, Faculty of Science and Technology, University of Debrecen, P.O. Box 400, H-4002 Debrecen, Hungary

[2]Institute of Nuclear Research (Atomki), P.O. Box 51, H-4001 Debrecen, Hungary

[3]Department of Mechanics, Materials and Structures, Budapest University of Technology and Economics, Műegyetem rkp. 3., K261, 1111 Budapest, Hungary

[4]MTA-BME Morphodynamics Reserarch Group, Műegyetem rkp. 3., K261, 1111 Budapest, Hungary
[*]Corresponding author Email: ferenc.kun@science.unideb.hu

1 Mathematical background

1.1 Invariant versus non-invariant formalism

Equation (4) of the main paper gave the formula for what is called the *curve-shortening flow*:

$$V = c\kappa \tag{S1}$$

where V denotes the propagation speed along the inward normal, c is a constant and κ is the signed curvature. Equation (S1) is written in an invariant notation, however, it can be also expanded into a notation using coordinates. The most convenient ones appear to be polar coordinates, i.e. we describe the curve as a scalar distance r measured from an arbitrarily picked interior point O and we parametrize r by the polar angle $\phi \in [0, 2\pi)$ and we seek the evolution in time t, given as a function $r(\phi, t)$.

The curvature κ may be expressed [46] as

$$\kappa(r, r_\phi, r_{\phi\phi}) = \frac{r^2 + 2r_\phi^2 - r r_{\phi\phi}}{(r^2 + r_\phi^2)^{\frac{3}{2}}}. \tag{S2}$$

where subscripts refer to partial derivatives. (Note that the above polar curvature formula was already known to Newton [44].) So we may write instead of equation (S1):

$$V = V(r, r_\phi, r_{\phi\phi}) = c\frac{r^2 + 2r_\phi^2 - r r_{\phi\phi}}{(r^2 + r_\phi^2)^{\frac{3}{2}}}. \tag{S3}$$

To turn this into a proper partial differential equation we have to transform inward normal speed V into radial speed r_t:

$$r_t = -V(r, r_\phi, r_{\phi\phi})w(r, r_\phi) \tag{S4}$$

where subscript t denotes partial derivative with respect to time t and

$$w = \sqrt{\frac{r^2 + r_\phi^2}{r^2}}. \tag{S5}$$

Using these formulae we may write instead of (S1):

$$r_t = -c\frac{r^2 + 2r_\phi^2 - r r_{\phi\phi}}{(r^2 + r_\phi^2)^{\frac{3}{2}}}\sqrt{\frac{r^2 + r_\phi^2}{r^2}}. \tag{S6}$$

Equation (S6) is the curve-shortening flow (S1) written az a proper partial differential equation for the function $r(\phi, t)$. Both types of notations have their advantages and disadvantages. An obvious advantage of (S1) is its compactness and intuitive appeal. On the olther hand, for numerical computations one would need either (S6) or some other, related formula. One obvious disadvantage of (S6) is that in includes the arbitrary choice of an origin. Not only does this make the formulae more complicated, we have to observe that, according to Grayson [20], (S1) shrinks arbitrary, closed curved to round points in finite time. So, *almost all* points which are *internal* at time $t = 0$ will become after finite time *external points* and as such, they will not be suitable to serve as origins for the coordinate system.

Based on these considerations, for the purposes of our current paper, describing global, qualitative features of the flow, the invariant formulation (S1) appears to be optimally suited.

We remark that in 3D the contrast is much more striking, formulae analogous to (S2) and (S6) are rather complex and thus it might be hard to

connect them intuitively with the physical process. Below we give the formula for the Gaussian curvature K expressed in general surface coordinates u, v:

$$
\begin{aligned}
K = {} & \Bigg(\Bigg(\Bigg(\frac{(\sin(v))^2 \left(2\left(\frac{\partial}{\partial u}R(u,v)\right)^2 - \left(\frac{\partial}{\partial v}R(u,v)\right)\cos(v)\,R(u,v)\sin(v) - R(u,v)\frac{\partial^2}{\partial u^2}R(u,v) + (R(u,v))^2 - (R(u,v))^2(\cos(v))^2\right)}{(R(u,v))^2 + \left(\frac{\partial}{\partial v}R(u,v)\right)^2 - (R(u,v))^2(\cos(v))^2 - \left(\frac{\partial}{\partial v}R(u,v)\right)^2(\cos(v))^2 + \left(\frac{\partial}{\partial u}R(u,v)\right)^2}\Bigg) \\
& \Bigg(\frac{\left(-R(u,v)\frac{\partial^2}{\partial v^2}R(u,v) + (R(u,v))^2 + 2\left(\frac{\partial}{\partial v}R(u,v)\right)^2\right)}{(R(u,v))^2 + \left(\frac{\partial}{\partial v}R(u,v)\right)^2 - (R(u,v))^2(\cos(v))^2 - \left(\frac{\partial}{\partial v}R(u,v)\right)^2(\cos(v))^2 + \left(\frac{\partial}{\partial u}R(u,v)\right)^2}\Bigg)\Bigg) - \\
& - \frac{\left(2\sin(v)\left(\frac{\partial}{\partial v}R(u,v)\right)\frac{\partial}{\partial u}R(u,v) - R(u,v)\left(\frac{\partial^2}{\partial v\partial u}R(u,v)\right)\sin(v) + R(u,v)\left(\frac{\partial}{\partial u}R(u,v)\right)\cos(v)\right)^2}{(R(u,v))^2 + \left(\frac{\partial}{\partial v}R(u,v)\right)^2 - (R(u,v))^2(\cos(v))^2 - \left(\frac{\partial}{\partial v}R(u,v)\right)^2(\cos(v))^2 + \left(\frac{\partial}{\partial u}R(u,v)\right)^2}\Bigg) \\
& \left(\left((R(u,v))^2 - (R(u,v))^2(\cos(v))^2 + \left(\frac{\partial}{\partial u}R(u,v)\right)^2\right)\left((R(u,v))^2 + \left(\frac{\partial}{\partial v}R(u,v)\right)^2\right) - \left(\frac{\partial}{\partial u}R(u,v)\right)^2\left(\frac{\partial}{\partial v}R(u,v)\right)^2\right)^{-1}
\end{aligned}
\tag{S7}
$$

Equation (S7) has been obtained by using Maple 16 and it is the 3D analogue of equation (S2). In sharp contrast, the invariant formulation analogous to equation (S1) is rather transparent in 3D:

$$V = cK. \tag{S8}$$

1.2 Some global properties of curvature-driven flows

The curvature-shortening flow (S1) belongs to a broad class of nonlinear partial differential equations (PDEs) called *curvature-driven flows* where the speed of evolution in the normal direction is given as some function of the curvature (in two dimensions) or curvatures (in higher dimensions). These equations are sometimes also referred to as *geometric heat equations* While locally defined, curvature-driven flows have startling global properties, e.g. they can shrink curves and surfaces to round points [17, 18, 19]. These features made these flows powerful tools to prove topological theorems which ultimately led, via their generalizations by Hamilton [21] to Perelman's celebrated proof [36] of the Poincaré conjecture. The global features of curvature-driven flows are mostly related to the monotonic change of quantities, such as the entropy associated with Gaussian curvature [8], other functionals, such as the Huisken functional [23] in case of the Mean Curvature flow, the number of critical points of the curvature (used in the Curvature Scale Space model for image processing [32, 33]), or the number of spatial critical points (with respect to a chosen reference point) [10, 19], which are closely related

to the geometry of the caustic [15, 5]. The curve-shortening flow (S1) and its 3D analogue, the Gauss curvature flow (S7) can be also viewed as geometric versions of the heat equation. They operate as *diffusion equations* where the conserved quantity is the curvature. They drive all curves (2D) and surfaces (3D) towards round points, i.e. their global attractor is the circle (2D) and the sphere (3D) characterized by constant curvature. This property can also be captured by considering the *curvature entropy* [8] analogous to the entropy associated with a random variable. Curvature entropy is a monotonically increasing in time under (S1) and (S8), reaching its absolute maximum as the curve approaches the circle and the surface approaches the sphere, respectively [8].

1.3 Curvature-driven flows as models of abrasion and the evolution of geophysical shape descriptors

Beyond offering powerful tools to prove mathematical statements, curvature-driven flows also have broad physical applications ranging from surface growth [24] through image processing [25, 30] to mathematical models of abrasion [4, 16]. Our paper is primarily motivated by the latter applications, where curvature-driven flows represent the fundamental model for the abrasion of pebbles under impacts of large particles. In particular, equation (S1) has been proposed by Firey [16] to model the extreme case of shape evolution of pebbles under collisions with infinitely large abraders and later it was shown [11] that (S1) remains an adequate geophysical model as the pebble collides with particles larger than its own size. Firey [16] proved convergence to the sphere under a symmetry assumption which was later eliminated by Andrews [2, 3].

While the rounding process is probably best captured by the monotonic evolution of the curvature entropy [8], measuring the latter and relating it to traditional geophysical shape descriptors is problematic. On the other hand, the dimensionless isoperimetric quotient (or circularity)

$$R = \frac{4\pi A}{P^2} \tag{S9}$$

(where A is the enclosed area, P is the length of the perimeter) is not only broadly used in geophysics, but also its monotonic evolution under (S1) has been proven [17].While the evolution of R has been used in field studies to verify curvature-driven flows as mathematical models of natural abrasion processes [40, 34], we are not aware of any previous attempt to compare measured or computed evolution $R(t)$ to a particle-based microscopic model.

The best established geophysical shape descriptors are, without doubt, dimensionless axis ratios [47]

$$y_1 = c/a, \qquad y_2 = b/a, \tag{S10}$$

where $a \geq b \geq c$ refer to the three characteristic orthogonal dimensions of the particle. For general shapes, a is taken as the largest diameter, b is taken as the largest diameter orthogonal to a and c is taken as the largest diameter orthogonal to both a and b. Alternatively, if the orthogonal bounding box with minimal volume is readily available, a,b and c are taken as the side length of this bounding box. Here we use the second interpretation but note that the two definitions do not, in general, coincide.

While axis ratios are well established in the geological community, there is little known about their evolution under (S1). Although it is clear that as the shape converges to the sphere, both y_1 and y_2 will approach 1, it is not known whether this happens in a monotonic manner. Still, axis ratios have been used in a range of field studies [34, 48] to record some aspects of the rounding process, however, direct comparisons between the PDE models and the field data was challenging as the former did not forecast any marked, specific feature for the evolution of the latter. The most direct comparison between axis ratio evolution predicted by (S1) and laboratory measurements has been conducted in [12], where cuboid samples were abraded in a steel drum. This highly targeted experiment was inspired by a spectacular mathematical result by Richard Hamilton [22] which we discuss below. Before laying out Hamilton's argument we also note that we are not aware of any previous attempt to compare the measured or computed evolution of axis ratios to a particle-based microscopic model.

1.4 Hamilton's result, the cuboid experiment and the evolution of the intact surface ratio

This close analogy between the curve-shortening flow (S1), its 3D version, the Gaussian flow (S7) and the 2D and 3D versions of the heat equation led to speculations whether a curious property of the latter, called *instant heat transfer* [14] might be also present in the curve-shortening flow and the Gaussian flow. The phenomenon of instant heat transfer can be summarized as follows: if we apply a concentrated heat source as initial condition to the heat equation, the latter predicts that heat will propagate at infinite speed, clearly contradicting the fundamental laws of physics. The conclusion is that instant heat transfer can be regarded as an artefact of the mathematical model, however, it was not clear that this deficit is also inherited

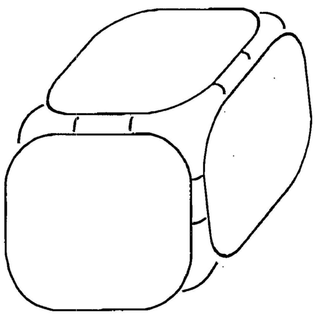

Figure 1: Hamilton's hand-drawn sketch [22] showing the initial phase of abrasion on a cuboid particle.

by curvature-driven flows. In the geometric flows concentrated heat source would translate into a vertex and instant heat transfer would correspond to instant, global smoothing, i.e. instant erosion of all flat areas. The presence or absence of this phenomenon promised, combined with the suitable choice for initial conditions, to be a well-observable, global geometric feature of natural abrasion. Richard Hamilton was first to note [22] that the Gaussian flow predicts finite diffusion speed for the curvature, and this implies that if the initial shape has *flat faces* (corresponding to zero temperature in the heat analogy) then some amount of flat area $S(t)$ will survive for a finite amount of time $t \leq t^*$ corresponding to some relative residual mass μ^*. This discovery suggested that studying the *intact surface ratio*

$$\gamma(t) = \frac{S(t)}{S(0)} \tag{S11}$$

may shed light on the fundamental features of shape evolution. Hamilton's result predicts that in the experiment with mildly elongated cuboids $\gamma(t)$ should decrease initially, drop to zero at some finite time $t = t^*$ corresponding to some relative residual mass $\mu = \mu^*$. Hamilton's result inspired further, detailed research into related curvature-driven PDE models. Together with Daskalopoulos he investigated this phenomenon in some detail [9]. Caputo and Daskalopoulos [6] considered the flow by the harmonic mean of the principal curvatures for surfaces which are initially uniformly convex with the exception of one flat side and showed that under this flow, the boundary of the flat side evolves by the curve shortening flow, so again flat sides persist for some time.

While Hamilton's result did not provide any general clue on the evolution of the axis ratios, however, it naturally lent itself to the design of a *specific experiment* in which the mathematical findings predicted well-observable qual-

itative features on the evolution both the axis ratios y_1, y_2 and the evolution $R(t)$ of the isoperimetric quotient. In fact,the basic idea of this experiment was suggested by Hamilton himself [22] who, at the end of his paper drew a sketch (see Figure 1) of a mildly elongated cuboid which was already abraded at the vertices and along the edges but still, some areas on the faces remained intact and he remarked that '*it would be fun to see a computer picture*'. The experiment thus suggested includes the abrasion of mildly elongated cuboid particles. This can be realized as a physical experiment where mildly elongated cuboid particles are put into a rotating steel drum. As we expect that (S8) is the governing equation for the geometric evolution, comparison of the experimental results with the numerical solution of (S8) appears to be reasonable. Hamilton's mathematical findings predicted (in this specific experiment, with the chosen initial shapes) axis ratios to be initially constant and then increase, on the other hand, isoperimetric ratio to increase initially and then have a plateau, suggesting the existence of two separated geometric phases: the first with constant axis ratios and increasing isoperimetric ratio and the second with constant isoperimetric ratio and increasing axis ratios.

This experiment (i.e. the physical experiment and comparison with the numerical simulation of the PDE) has been performed and in [12] it was reported that it met the expectations, moreover, the evolutions $y_1(t), y_2(t)$ and $R(t)$ clearly marked two *geometric phases*: in the first phase axis ratios remained approximately constant and the isoperimetric quotient increased while in the second phase the opposite happened: axis ratios increased while the isoperimetric quotient remained approximately constant. These two geometric phases were separated by a rapid transition at the residual relative mass $\mu^* \approx 0.34$. While these findings certainly vindicated Hamilton's result on an experimental level, one step remained clearly missing: establishing a connection between an element-based simulation and the partial differential equation (S8), i.e. to find the link between the underlying physics of fragmentation and the mean field geometric models. This gap remained significant, as only such a DEM simulation had the potential to delineate the energy regime in which the mean-field theory can be applied. *The cuboid experiment is also at the core of this paper* where we establish this missing link. We use this very special, targeted experiment for the following reasons:

1. this is the only known experiment where a direct, verifiable prediction can be made both on the evolution $y_1(t), y_2(t)$ of axis ratios and the evolution $R(t)$ isoperimetric quotient,

2. this is the only known experiment where the above evolutions have already been recorded in a physical experiment and in a direct numerical simulation of the PDE and

3. in this experiment the DEM simulations admit the recording of the evolution $\gamma(t)$ of the remaining intact surface ratio which can reveal an additional, more direct confirmation of Hamilton's result.

As we point out in the main text, the DEM simulation met our expectations:

1. We identified the two geometric phases separated at $\mu^* = 0.34$ in the evolutions $y_1(t), y_2(t), R(t)$ in perfect agreement with the data reported in [12],

2. we computed the evolution $\gamma(t)$ which also showed a sharp two-phase behaviour: $\gamma(t)$ dropped to zero at residual mass $\mu^* = 0.34$ which agreed with the value separating the phases of axis ratio and isoperimetric quotient evolution.

3. Finally, we uncovered that this behaviour is characteristic only for one *energy phase* and we identified this phase.

The listed findings establish the link between the geometric mean-field theory represented by the partial differential equation (S8) and a particle-based discrete element model and thus serve as a foundation to use the former as legitimate abrasion models in the energy range identified in our paper.

2 Discrete element model

Here we provide a detailed description of the construction of the discrete element model including also the values of the most important material's parameters used in the computer simulations.

2.1 Model construction

We performed computer simulations of the repeated sub-critical impact of solid bodies against a hard wall in the framework of a discrete element model of heterogeneous materials which has been successfully applied before to investigate fracture and fragmentation under various types of loading conditions. In the model, the impactor is assumed to have an initial angular shape which was modeled by constructing rectangular bodies of mildly elongated cubic shape with the aspect ratio $1 : 1.2 : 1.4$ of their shortest c_0, intermediate b_0, and longest a_0 sides. The cubic sample is represented as a random packing of spherical particles which was generated by sedimenting particles in a rectangular container [28, 29, 27]. Material's heterogeneity is captured by the randomness of the particle diameter d, which was sampled from a

uniform distribution in a narrow range $\Delta d/\langle d\rangle = 0.05$, where $\langle d\rangle$ denotes the average particle diameter. The total number of particles in the samples fluctuates around 12.000. Cohesive interaction is realized by beam elements which connect the particles along the edges of Delaunay triangles constructed from the initial particle positions [28, 29, 27].

During the impact process in three dimensions (3D) the total deformation of a beam is calculated as the superposition of elongation, torsion, as well as, bending and shearing [7, 45]. Cracks are formed when overstressed beams break according to a physical breaking rule. The breaking condition takes into account the stretching and shearing of particles contacts

$$\left(\frac{\varepsilon_{ij}}{\varepsilon_{th}}\right)^2 + \frac{\max(\Theta_i, \Theta_j)}{\Theta_{th}} \geq 1. \tag{S12}$$

Here ε_{ij} denotes the axial strain of the beam between particles i and j, while Θ_i, and Θ_j are the bending angles of the beam ends. The parameters ε_{th} and Θ_{th} control the relative importance of the two breaking modes [26, 7, 42, 29, 28]. The breaking criterion is evaluated after each iteration step and those beams which fulfill the condition are removed from the system. In the model there is only structural disorder present, i.e. the breaking thresholds are constant $\varepsilon_{th} = 0.02$ and $\Theta_{th} = 3^o$, however, the physical properties of beams such as length, cross section, and elastic moduli, are determined by the random particle packing. At the broken beams along the surface of the spheres cracks are generated inside the solid and as a result of the successive beam breaking the solid falls apart. The interaction of those particles which are not connected by beams, e.g. because the beam has been broken, is described by the Hertz contact law [37]. The Hertz contact ensures that force can be transmitted through crack faces when they are pressed against each other. The time evolution of the impacting body is generated by solving the equation of motion of all the particles. For the numerical solution a fifth order Predictor-Corrector scheme is used which provides a sufficient accuracy of the calculations [1]. To ensure numerical stability and slightly enhance dissipation of internal vibrations of fragments a weak viscous type damping force is added to the inter-particle forces [37] which do not have any significant effect on the overall time evolution of the impact process.

To study how the shape of freshly fractured rock bodies evolves through repeated low velocity collisions, the initial geometry of the impacting solid was fixed in the simulations to be a cuboid. This choice has two motivations, on the one hand, the cubic shape is the simplest 3D object which captures the sharp corners and edges of real rock pieces, and on the other hand, recently we have shown that the average shape of fragments is well approximated by a cube for a broad class of fragmentation processes from the breakup of rock

Parameters of the discrete element model			
**Beams:**			
longitudinal stiffness	E^b	6	GPa
strain threshold	ε_{th}	0.02	-
bending threshold	θ_{th}	3	*degree*
**Particles:**			
stiffness	E^p	3	GPa
Average diameter	$\langle d \rangle$	0.5	mm
density	ρ	3000	kg/m^3
**Hard wall:**			
stiffness	E^w	70	GPa
**Interaction:**			
friction coefficient	μ	1	-
damping coefficient (normal)	γ_n	0.25	s^{-1}
friction coefficient (tangential)	γ_t	0.05	s^{-1}
**System:**			
time increment	Δt	1e-7	s
average number of particles	N^p	12000	-
average number of beams	N^b	105000	-
solid fraction		0.65	-
**Macroscopic properties (DEM):**			
system stiffness	E	7.4 ± 0.5	GPa
Poisson's ratio	ν	0.2	-
system strength	σ_c	110	MPa

Table S1: Micro- and macroscopic material properties of numerical samples.

walls through sequential cracking to the rapid disintegration of rocks in an explosion [13].

2.2 Parameters used in the simulations

The values of the most important parameters of the model used throughout the computer simulations are summarized in Table S1.

An important element of our approach is that the parameters of the model are not fitted to any specific rock material. Instead, material and breaking characteristics are set to form a generic model which provides a consistent qualitative, and in certain cases quantitative description of the mechanical

and fracture properties of the broad class of heterogeneous brittle materials. This class of materials is abundant in our geological environment [43], hence, they are in the focus of all theoretical and experimental studies on shape evolution. The consistency of the model has been carefully tested before (1) by comparing the stress field emerging inside the solid during impact with a hard wall to finite element calculations [7, 45], and (2) by comparing the temporal evolution of cracking, the spatial structure of the crack pattern, and the statistics of fragments to a large number of laboratory experiments supported by high speed imaging [31, 38, 39]. The model material in the simulations (see Table S1) has a lower stiffness and a lower breaking strength than real rocks, which is typical for DEM studies, since simulations with realistic Young moduli would not be feasible.

In our former studies of the fragmentation of heterogeneous brittle materials in the range of impact velocities $v_0 > v_f$, our DEM provided power law distribution of fragment masses with exponents in quantitative agreement with laboratory measurements [7, 42, 35]. Most importantly, the model made it also possible to deduce scaling laws of fragmentation phenomena and to determine the value of the critical exponents of the damage-fragmentation phase transition [7, 42, 35, 41]. In our present study, the same material parameters were used as in the above carefully tested cases.

References

[1] M. P. Allen and D. J. Tildesley. *Computer simulation of Liquids.* Clarendon Press, Oxford, 2002.

[2] B. Andrews. Contraction of convex hypersurfaces in Euclidean space. *Cal. Var.*, 2:151–171, 1994.

[3] B. Andrews. Gauss curvature flow: the fate of rolling stones. *Invet. Math*, 138:151–161, 1999.

[4] F. J. Bloore. The shape of pebbles. *Math. Geol.*, 9:113–122, 1977.

[5] J. W. Bruce, P. J. Giblin, and C. G. Gibson. On caustics of plane curves. *Amer. Math. Monthly*, 88:651–667, 1981.

[6] M. Caputo and P. Daskalopoulos. Highly degenerate harmonic mean curvature flow. *Calc. Var. Partial Differential Equations*, 35(3):365–384, 2009.

[7] H. A. Carmona, F. K. Wittel, F. Kun, and H. J. Herrmann. Fragmentation processes in impact of spheres. *Phys. Rev. E*, 77:051302, 2008.

[8] B. Chow. On Harnack's inequailty and entropy for the Gaussian curvature flow. *Comm. Pure and Applied Math.*, XLIV:469–483, 1991.

[9] P. Daskalopoulos and R. Hamilton. The free boundary in the gauss curvature flow with flat sides. *J. Reine Angew. Math.*, 510:187–227, 1999.

[10] G. Domokos. Monotonicity of Spatial Critical Points Evolving Under Curvature-Driven Flows. *Journal of Nonlinear Science*, 25(2):247–275, apr 2015.

[11] G. Domokos and G. W. Gibbons. The evolution of pebble size and shape in space and time. *Proc. Roy. Soc. A.*, 468:3059–3079, 2012.

[12] G. Domokos, D. Jerolmack, A. A. Sipos, and A. Török. How river rocks round: explinaining the size-shape paradox. *PloS One*, DOI:10.1371/journal.pone.0088657, 2014.

[13] G. Domokos, D. J. Jerolmack, F. Kun, and J. Török. Plato's cube and the natural geometry of fragmentation. *Proceedings of the National Academy of Sciences*, 117(31):18178–18185, 2020.

[14] L. Evans. *Partial Differential Equations, 2nd edition.* American Mathematical Society, 2010.

[15] D. L. Fidal and P. J. Giblin. Generic one-parameter families of caustics in the plane. *Math. Proc. Camb. Philos. Soc.*, 96:425–432, 1984.

[16] W. Firey. Shapes of worn stones. *Mathematika*, 21(1):1–11, 1974.

[17] M. Gage. An isoperimetric inequality with applications to curve shortening. *Duke Math. J.*, 50:1225–1229, 1987.

[18] M. Gage and R. Hamilton. The heat equation shrinking convex plane curves. *J. Differ. Geom.*, 23:69–96, 1986.

[19] M. Grayson. The heat equation shrinks embedded plane curves to round points. *J. Differ. Geom.*, 26:285–314, 1987.

[20] M. A. Grayson. The heat equation shrinks embedded plane curves to round points. *J. Diff. Geom.*, 26:285–314, 1987.

[21] R. Hamilton. Three-manifolds with positive Ricci curvature. *J. Diff. Geom.*, 17:255–306, 1982.

[22] R. Hamilton. Worn stones with flat sides. *Discourses Math. Appl.*, 3:69–78, 1994.

[23] G. Huisken. Flow by mean curvature of convex surfaces into spheres. *J. Diff. Geom.*, 20:237–266, 1984.

[24] M. Kardar, G. Parisi, and Y.-C. Zhang. Dynamic scaling of growing interfaces,. *Phys. Rev. Letters*, 56:889–892, 1986.

[25] J. Koenderink. The structure of images. *Biol. Cybern.*, 50:363–370, 1984.

[26] F. Kun and H. J. Herrmann. A study of fragmentation processes using a discrete element method. *Comp. Meth. Appl. Mech. Eng.*, 138:3, 1996.

[27] F. Kun, G. Pl, I. Varga, and I. G. Main. Effect of disorder on the spatial structure of damage in slowly compressed porous rocks. *Philosophical Transactions of the Royal Society A: Mathematical, Physical and Engineering Sciences*, 377(2136):20170393, 2019.

[28] F. Kun, I. Varga, S. Lennartz-Sassinek, and I. G. Main. Approach to failure in porous granular materials under compression. *Phys. Rev. E*, 88:062207, 2013.

[29] F. Kun, I. Varga, S. Lennartz-Sassinek, and I. G. Main. Rupture cascades in a discrete element model of a porous sedimentary rock. *Phys. Rev. Lett.*, 112:065501, 2014.

[30] C. Lu, Y. Cao, and D. Mumford. Surface evolution under curvature flows. *J. Visual Communication and Imgae Representation*, 13:65–81, 2002.

[31] R. Majzoub and M. Chaudhri. High-speed photography of low-velocity impact cracking of solid spheres. *Phil. Mag. Lett.*, 80(6):387–393, 2000.

[32] F. Mokhtarian, S. Abbasi, and J. Kittler. Efficient and robust retrieval by shape content through curvature scale space. In *International Workshop on Image Databases and Multimedia Search*, pages 35–42, 1996.

[33] F. Mokhtarian and R. Suomela. Robust image corner detection through curvature scale space. *Pattern Analysis and Machine Intelligence, IEEE Transactions on*, 20(12):1376–1381, Dec 1998.

[34] T. Novk-Szabó, A. A. Sipos, S. Shaw, D. Bertoni, A. Pozzebon, E. Grottoli, G. Sarti, P. Ciavola, G. Domokos, and D. J. Jerolmack. Universal characteristics of particle shape evolution by bed-load chipping. *Science Advances*, 4(3):eaao4946, Mar. 2018.

[35] G. Pál, I. Varga, and F. Kun. Emergence of energy dependence in the fragmentation of heterogeneous materials. *Phys. Rev. E*, 90:062811, 2014.

[36] G. Perelman. Ricci flow with surgery on three-manifolds. *http://arXiv.org/math.DG/0303109v1*, 2003.

[37] T. Pöschel and T. Schwager. *Computational Granular Dynamics*. Springer, Berlin, 2005.

[38] A. Salman, C. Biggs, J. Fu, I. Angyal, M. Szabó, and M. Hounslow. An experimental investigation of particle fragmentation using single particle impact studies. *Powder Technology*, 128:36 – 46, 2002.

[39] K. Schönert. Breakage of spheres and circular discs. *Powder Technology*, 143-144:2 – 18, 2004.

[40] T. Szab, G. Domokos, J. P. Grotzinger, and D. J. Jerolmack. Reconstructing the transport history of pebbles on Mars. *Nature Communications*, 6:8366, 2015.

[41] G. Timár, J. Blömer, F. Kun, and H. J. Herrmann. New universality class for the fragmentation of plastic materials. *Phys. Rev. Lett.*, 104:095502, 2010.

[42] G. Timár, F. Kun, H. A. Carmona, and H. J. Herrmann. Scaling laws for impact fragmentation of spherical solids. *Phys. Rev. E*, 86:016113, 2012.

[43] D. L. Turcotte. *Fractals and chaos in geology and geophysics*. Cambridge University Press, 1997.

[44] D. Whiteside, editor. *The Mathematical Papers of Isaac Newton, Vol 3*. Cambridge University Press, 1969.

[45] F. Wittel, H. Carmona, F. Kun, and H. Herrmann. Mechanisms in impact fragmentation. *Int J Fract*, 154:105, 2008.

[46] E. Zeidler, editor. *Oxford's User Guide to Mathematics.p 772*. Oxford University Press, Oxford, New York, 2004.

[47] T. Zingg. Beiträge zur schottenanalyse. *Schweiz Mineral Petrogr Mitt*, 15:39–140, 1935.

[48] T. Zingg. Size grading along a pebble beach: Chesil beach, england. *J. Sediment. Petr.*, 39:297–311, 1969.
